# Longitudinal Effectiveness of Repeated Lifestyle Education in Pediatric Dyslipidemia: Developmental and Environmental Modifiers in a Real-World Clinical Cohort

**DOI:** 10.3390/children13050682

**Published:** 2026-05-16

**Authors:** Sung Yong Min, Eun Young Kim

**Affiliations:** 1 Department of Pediatrics, Chosun University Hospital, Gwangju 61453, Republic of Korea; symin@csuh.co.kr; 2 Department of Pediatrics, Chosun University College of Medicine, Gwangju 61452, Republic of Korea

**Keywords:** pediatric dyslipidemia, lifestyle intervention, lipid profile, longitudinal analysis, puberty, birth weight, metabolic risk

## Abstract

Background/Objectives: Pediatric dyslipidemia is a major modifiable risk factor for future cardiovascular disease, and lifestyle modification is recommended as first-line therapy. However, real-world longitudinal evidence on the effectiveness of repeated lifestyle education delivered during routine clinical practice remains limited. In this study, we assessed longitudinal metabolic changes following repeated lifestyle education and explored developmental and early-life factors associated with treatment responsiveness. Methods: In this retrospective longitudinal cohort study, we included 437 children and adolescents newly diagnosed with dyslipidemia at a tertiary hospital between 2019 and 2024. Participants received repeated lifestyle education during routine outpatient visits. Anthropometric and laboratory parameters were assessed over time. Linear mixed models were used to evaluate longitudinal changes, and multivariable logistic regression analyses were performed to identify predictors of lipid improvement. Results: Repeated lifestyle education was associated with gradual improvements in BMI SDS, total cholesterol, and non-HDL cholesterol over time. Linear mixed model analyses demonstrated significant time effects for total cholesterol and non-HDL cholesterol, while HDL cholesterol remained relatively stable. Thyroid-stimulating hormone (TSH) also demonstrated a significant time-dependent reduction during follow-up. Multivariable logistic regression analysis revealed that pubertal stage was associated with a lower likelihood of improvement in LDL and non-HDL cholesterol, whereas large-for-gestational-age birth was associated with a higher likelihood of HDL improvement. Conclusions: Repeated lifestyle education delivered during routine clinical practice was associated with meaningful improvements in lipid profiles in children with dyslipidemia. Developmental stage and early-life characteristics may influence treatment responsiveness, highlighting the importance of individualized and developmentally informed management strategies.

## 1. Introduction

Dyslipidemia during childhood has emerged as an important predictor of cardiovascular disease later in life [1,2,3]. Accumulating evidence indicates that lipid abnormalities identified in pediatric populations often persist into adulthood and contribute to early atherosclerotic changes [4,5]. Early detection and management of abnormal lipid profiles therefore represent a key strategy for long-term cardiovascular risk prevention.

Lifestyle modification remains the cornerstone of treatment for pediatric dyslipidemia. Current guidelines emphasize dietary modification, increased physical activity, and reduction of sedentary behaviors as first-line interventions before pharmacologic therapy is considered [6,7,8]. Despite these recommendations, most existing studies evaluating lifestyle interventions in pediatric dyslipidemia have been conducted in controlled research settings. Consequently, relatively little is known about the effectiveness of repeated lifestyle education delivered in routine outpatient clinical practice.

In real-world clinical settings, lifestyle counseling is typically provided repeatedly over multiple visits rather than as a single structured intervention. These repeated encounters may play an important role in reinforcing behavioral changes and supporting sustained metabolic improvement [7,8]. However, longitudinal clinical data evaluating the cumulative impact of repeated lifestyle education remain limited.

In addition to lifestyle behaviors, metabolic responses to intervention may also be influenced by developmental and early-life factors. Pubertal maturation is accompanied by substantial hormonal and metabolic changes that affect lipid metabolism, insulin sensitivity, and body composition [9,10]. Furthermore, increasing evidence suggests that early-life growth patterns, including birth weight, may influence long-term metabolic regulation through developmental programming mechanisms [11,12,13].

Understanding how these developmental and environmental factors interact with lifestyle interventions may help identify children who are more likely to benefit from non-pharmacologic management strategies.

The present study therefore aimed to evaluate the longitudinal effectiveness of repeated lifestyle education in children and adolescents newly diagnosed with dyslipidemia in a real-world outpatient setting. In addition, we sought to identify developmental and early-life factors associated with lipid improvement during follow-up.

## 2. Materials and Methods

### 2.1. Study Population

This retrospective cohort study included children and adolescents who visited the pediatric outpatient clinic of Chosun University Hospital between January 2019 and December 2024 and were newly diagnosed with dyslipidemia during this period. Patients were eligible for inclusion if they underwent fasting lipid testing at the time of diagnosis and had at least one follow-up visit with repeated laboratory evaluation after receiving lifestyle education. To explore potential environmental influences on metabolic outcomes, participants were categorized into three groups according to the time of diagnosis corresponding to distinct phases of the COVID-19 pandemic in Korea: the pre-pandemic period (2019), representing the period prior to the first confirmed COVID-19 case in Korea (January 2020); the early-pandemic period (2020–June 2021), characterized by nationwide school closures beginning in March 2020 and strict social distancing mandates that substantially disrupted children’s physical activity and dietary behaviors; and the late-pandemic/recovery period (July 2021–2024), corresponding to the gradual resumption of in-person schooling and the progressive relaxation of public health restrictions under Korea’s staged transition policy implemented from late 2021 onward [14]. Patients were excluded if they had previously diagnosed diabetes mellitus, thyroid disease requiring treatment, chronic systemic illness, or were receiving medications known to affect lipid metabolism. Patients with a history of precocious puberty or those undergoing growth hormone therapy were also excluded. After applying these criteria, 437 patients were included in the final analysis.

This study was approved by the Institutional Review Board of Chosun University Hospital (IRB No. 2025-08-005) on 13 August 2025. Because of the retrospective design, informed consent was waived and all data were anonymized prior to analysis.

### 2.2. Follow-Up Structure

Baseline evaluation was defined as the visit at which dyslipidemia was first diagnosed (T1). Subsequent visits were categorized as the first follow-up visit (T2), second follow-up visit (T3), and third follow-up visit (T4) when available. Follow-up visits were conducted as part of routine outpatient clinical care, typically at intervals of approximately 1–3 months, depending on individual clinical circumstances. Because this study reflects real-world clinical practice, follow-up intervals were not strictly standardized.

### 2.3. Anthropometric and Laboratory Measurements

Anthropometric measurements were obtained by trained nurses using standardized equipment. Height was measured using a Harpenden stadiometer (Holtain Ltd., Crymych, UK), and weight was measured using a calibrated digital scale (Seca GmbH & Co. KG, Hamburg, Germany). Body mass index (BMI) was calculated as weight divided by height squared (kg/m^2^), and BMI standard deviation scores (BMI SDS) were derived using the 2017 Korean National Growth Charts. Pubertal development was assessed by pediatric endocrinologists according to Tanner staging at every outpatient visit as part of routine clinical practice. For statistical modeling, only the baseline Tanner stage (T1) was used as a time-invariant covariate, as the primary research question concerned whether pubertal status at the time of diagnosis modified the lipid response to lifestyle education, rather than whether within-individual pubertal progression during follow-up mediated lipid change. This approach is consistent with standard practice in the pediatric dyslipidemia literature [8,10]. Blood samples were collected after an overnight fast of at least 8 h following the evening meal. Participants and caregivers were instructed to skip breakfast on the morning of blood sampling; a strict 12-h minimum was not required given the practical constraints of a pediatric outpatient setting [8]. Laboratory measurements included total cholesterol, triglycerides, HDL cholesterol, LDL cholesterol, fasting glucose, AST, ALT, thyroid-stimulating hormone, and free thyroxine. LDL cholesterol was calculated using the Friedewald equation (LDL = total cholesterol − HDL cholesterol − triglycerides/5) for all participants, as triglyceride levels were below 400 mg/dL in all cases. Non-HDL cholesterol was calculated by subtracting HDL cholesterol from total cholesterol.

### 2.4. Lifestyle Education

At each outpatient visit, patients and their caregivers received lifestyle counseling focusing on dietary modification and physical activity. Nutritional counseling was based on CHILD-1 dietary recommendations and current pediatric dyslipidemia management guidelines [6,7,8]. Counseling emphasized reduction of saturated fat intake, increased consumption of fruits and vegetables, and maintenance of balanced portion sizes appropriate for age. Exercise counseling encouraged regular moderate-to-vigorous physical activity and reduction of sedentary behaviors such as excessive screen time. Because this study was conducted as a retrospective real-world clinical cohort, adherence to lifestyle recommendations was not systematically documented using standardized instruments or scales. Accordingly, adherence could neither be quantitatively nor qualitatively assessed in a structured manner, and this represents an important limitation of the present study.

### 2.5. Definitions

Dyslipidemia was defined according to pediatric lipid management guidelines [7,8]. Abnormal lipid levels were defined as total cholesterol ≥ 200 mg/dL, LDL cholesterol ≥ 130 mg/dL, non-HDL cholesterol ≥ 145 mg/dL, triglycerides ≥ 100 mg/dL for children younger than 10 years or ≥130 mg/dL for those aged 10 years and older, and HDL cholesterol < 40 mg/dL. Birth weight was categorized as small-for-gestational-age (SGA), appropriate-for-gestational-age (AGA), or large-for-gestational-age (LGA) according to national reference standards.

### 2.6. Definition of Lipid Improvement

To identify predictors of treatment response, clinically meaningful lipid improvement between baseline and first follow-up was defined as an increase in HDL cholesterol of ≥5 mg/dL, a decrease in LDL cholesterol of ≥10 mg/dL, or a decrease in non-HDL cholesterol of ≥10 mg/dL. These thresholds were established a priori as exploratory definitions informed by established pediatric lipid management guidelines and prior intervention studies. The NHLBI Expert Panel (2011) noted that LDL cholesterol levels in children and adolescents may decrease by 10–20% or more with lifestyle modification, and the Dietary Intervention Study in Children (DISC) reported an LDL reduction of approximately 4.8 mg/dL after one year of dietary intervention [8,10]; our LDL threshold of ≥10 mg/dL therefore represents a conservative criterion. The HDL threshold of ≥5 mg/dL was selected as a conservative, clinically detectable increment, given the modest and variable effects of lifestyle modification on HDL cholesterol reported in pediatric intervention studies [15,16]. Because these were pre-specified exploratory criteria rather than validated clinical endpoints, sensitivity analyses using percentage-change definitions (HDL increase ≥ 10%, LDL decrease ≥ 10%, non-HDL decrease ≥ 10%) were additionally performed and are presented in Supplementary Table S4. To further examine whether the observed improvements were sustained over time, the persistence of lipid improvement at T4 was assessed using the more stringent percentage-change criteria (HDL increase ≥ 10%, LDL decrease ≥ 10%, non-HDL decrease ≥ 10%) among participants with available data (*n* = 111), and is reported in Supplementary Table S5.

### 2.7. Statistical Analysis

Continuous variables are presented as mean ± standard deviation, and categorical variables are expressed as frequencies and percentages. Baseline differences between groups were evaluated using one-way analysis of variance and chi-square test, with Bonferroni correction applied for post-hoc pairwise comparisons among pandemic-period groups. Longitudinal metabolic trajectories were analyzed using linear mixed models, adjusting for sex, birth weight category, family history of dyslipidemia, and Tanner stage at baseline (T1) as time-invariant covariates, and BMI SDS as a time-varying covariate updated at each visit (T1 through T4). Post-hoc pairwise comparisons among pandemic-period groups in the linear mixed model analyses were adjusted using the Bonferroni method. However, no correction was applied across the multiple outcome variables examined simultaneously; accordingly, findings from the linear mixed model analyses should be interpreted as exploratory and hypothesis-generating rather than confirmatory. Linear mixed models were estimated under the missing-at-random (MAR) assumption, which allows all participants with at least one available observation to contribute to the analysis without imputation. For AST, the unstructured (UN) covariance structure produced non-convergent variance estimates (SE ≈ mean; 95% CI extending into physiologically implausible negative values; ΔAIC vs. Compound Symmetry = −237.5), indicating severe overparameterization; accordingly, a compound symmetry (CS) covariance structure was applied for AST only, while the UN structure was retained for all other outcomes. Predictors of lipid improvement were evaluated using multivariable logistic regression analysis, and results are presented as odds ratios with 95% confidence intervals. A *p*-value < 0.05 was considered statistically significant. All statistical analyses were performed using IBM SPSS Statistics for Windows, Version 21.0 (IBM Corp., Armonk, NY, USA).

## 3. Results

### 3.1. Baseline Clinical Characteristics

A total of 437 children and adolescents newly diagnosed with dyslipidemia were included in this study. The mean age at diagnosis was 103.7 ± 20.7 months, and 65.7% of participants were female. A family history of dyslipidemia was present in 59.7% of participants. The mean BMI was 19.02 ± 3.78 kg/m^2^, and the mean BMI SDS was 0.61 ± 1.48. Approximately 17.8% of participants were classified as overweight or obese. The mean bone age at diagnosis was 9.07 ± 2.44 years. Birth weight averaged 3.14 ± 0.49 kg, with 4.8% of participants classified as small for gestational age and 8.0% as large for gestational age. Tanner stage II–V was observed in 62.3% of participants. Baseline lipid levels were as follows: total cholesterol 198.1 ± 24.6 mg/dL, triglyceride 121.2 ± 73.9 mg/dL, HDL cholesterol 56.3 ± 13.0 mg/dL, LDL cholesterol 124.4 ± 24.4 mg/dL, and non-HDL cholesterol 141.9 ± 23.3 mg/dL.

When participants were categorized according to time period, significant differences were observed in sex distribution, family history of dyslipidemia, and bone age at diagnosis. Female participants and those with a positive family history of dyslipidemia were more prevalent in the earlier time-period groups. Bone age at diagnosis was significantly higher in the earlier periods than in the later period. Other baseline anthropometric and lipid parameters did not differ significantly among groups (Table 1).

### 3.2. Longitudinal Changes Following Repeated Lifestyle Education

Repeated lifestyle education was associated with significant improvement in anthropometric and lipid parameters during follow-up. BMI SDS decreased significantly at both the first and second follow-up visits compared with baseline (Δ −0.11 ± 0.38, *p* < 0.001; Δ −0.09 ± 0.50, *p* = 0.010). Total cholesterol demonstrated significant reductions during follow-up (Δ −2.54 ± 21.99 mg/dL, *p* = 0.020 at first follow-up; Δ −5.15 ± 21.46 mg/dL, *p* < 0.001 at second follow-up). Similarly, non-HDL cholesterol showed significant decreases (Δ −2.04 ± 19.48 mg/dL, *p* = 0.030; Δ −4.05 ± 20.38 mg/dL, *p* < 0.001). Triglyceride and LDL cholesterol levels demonstrated decreasing trends during follow-up; however, these changes did not reach statistical significance. HDL cholesterol showed a slight reduction without statistical significance. These longitudinal changes in lipid parameters are summarized in Table 2.

### 3.3. Longitudinal Metabolic Changes Identified by Linear Mixed Model Analysis

Linear mixed model analysis demonstrated significant time-dependent reductions in total cholesterol and non-HDL cholesterol after adjusting for sex, family history of dyslipidemia, birth weight category, Tanner stage, and BMI SDS (Table 3). TSH levels also demonstrated significant time-dependent reductions. AST levels remained within normal laboratory reference ranges across all groups and time points (Supplementary Table S6).

**Figure 1:**
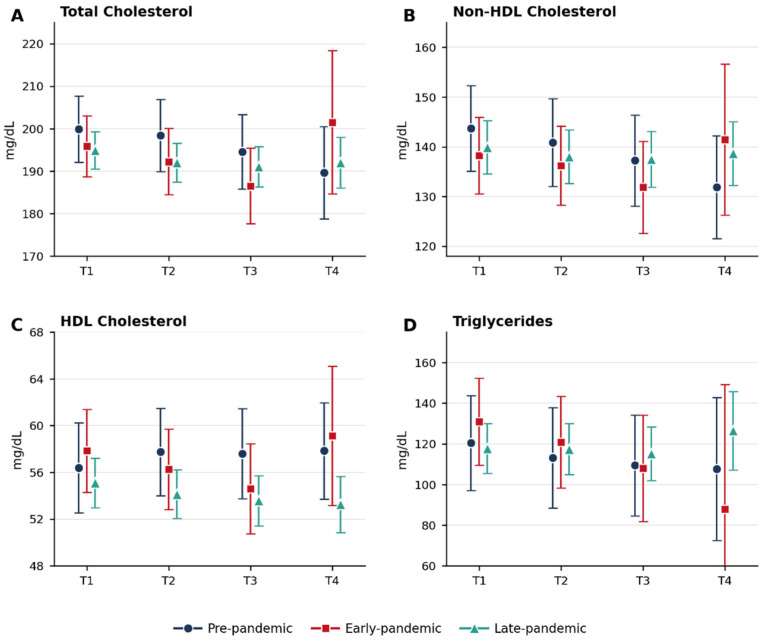
Longitudinal trajectories of lipid parameters during follow-up. Estimated marginal means with 95% confidence intervals derived from linear mixed models are shown for (**A**) total cholesterol, (**B**) non-HDL cholesterol, (**C**) HDL cholesterol, and (**D**) triglycerides. Time points represent baseline (T1) and subsequent follow-up visits (T2–T4). Groups correspond to COVID-19 pandemic phases defined in the Methods: Pre-pandemic (2019), Early-pandemic (2020–June 2021), and Late-pandemic (July 2021–2024). Models were adjusted for sex, family history of dyslipidemia, birth weight category, and Tanner stage (time-invariant), and BMI SDS at each visit (time-varying); estimated marginal means are presented at the mean BMI SDS value (0.529). Post-hoc pairwise comparisons among groups were adjusted using the Bonferroni method. Wide confidence intervals for the early-pandemic group at T4, particularly for triglycerides, reflect the markedly reduced sample size at that time point (*n* = 6) and should be interpreted with caution. Fixed-effects *p*-values from the linear mixed models were as follows—(**A**) Total Cholesterol: Time *p* = 0.011, Group *p* = 0.660, Time × Group *p* = 0.310; (**B**) Non-HDL Cholesterol: Time *p* = 0.037, Group *p* = 0.909, Time × Group *p* = 0.260; (**C**) HDL Cholesterol: Time *p* = 0.380, Group *p* = 0.037, Time × Group *p* = 0.250; (**D**) Triglycerides: Time *p* = 0.279, Group *p* = 0.681, Time × Group *p* = 0.649.

The longitudinal trajectories of major lipid parameters are illustrated in Figure 1. Total cholesterol and non-HDL cholesterol showed gradual reductions during follow-up across all groups, whereas HDL cholesterol demonstrated relatively stable patterns. Triglyceride levels showed variable trajectories across groups, with the early-pandemic group showing a greater decline at T3 followed by a notable rebound at T4; however, these fluctuations should be interpreted with caution, as the early-pandemic group had markedly reduced data availability at T4 (*n* = 6), resulting in wide confidence intervals that preclude reliable interpretation of the group-specific triglyceride trajectory at that time point.

### 3.4. Predictors of Lipid Improvement

Logistic regression analysis was performed to identify predictors of lipid improvement between baseline and first follow-up (Table 4). Participants in Tanner stage II–V showed reduced likelihood of LDL cholesterol improvement (odds ratio [OR] 0.61, 95% confidence interval [CI] 0.38–0.95, *p* = 0.030) and non-HDL cholesterol improvement (OR 0.60, 95% CI 0.38–0.96, *p* = 0.030) compared with prepubertal participants. Participants born large for gestational age demonstrated increased likelihood of HDL cholesterol improvement (OR 2.19, 95% CI 1.05–4.59, *p* = 0.040). Notably, baseline HDL cholesterol did not differ significantly across birth weight categories (SGA: 56.0 ± 14.9 mg/dL, AGA: 56.6 ± 13.0 mg/dL, LGA: 52.9 ± 12.2 mg/dL; one-way ANOVA *p* = 0.277), and the LGA group had numerically lower baseline HDL than the AGA group, suggesting that the observed advantage in HDL improvement was not attributable to higher baseline levels. Other variables, including sex, BMI SDS, family history, and time period, were not significantly associated with lipid improvement.

**Figure 2:**
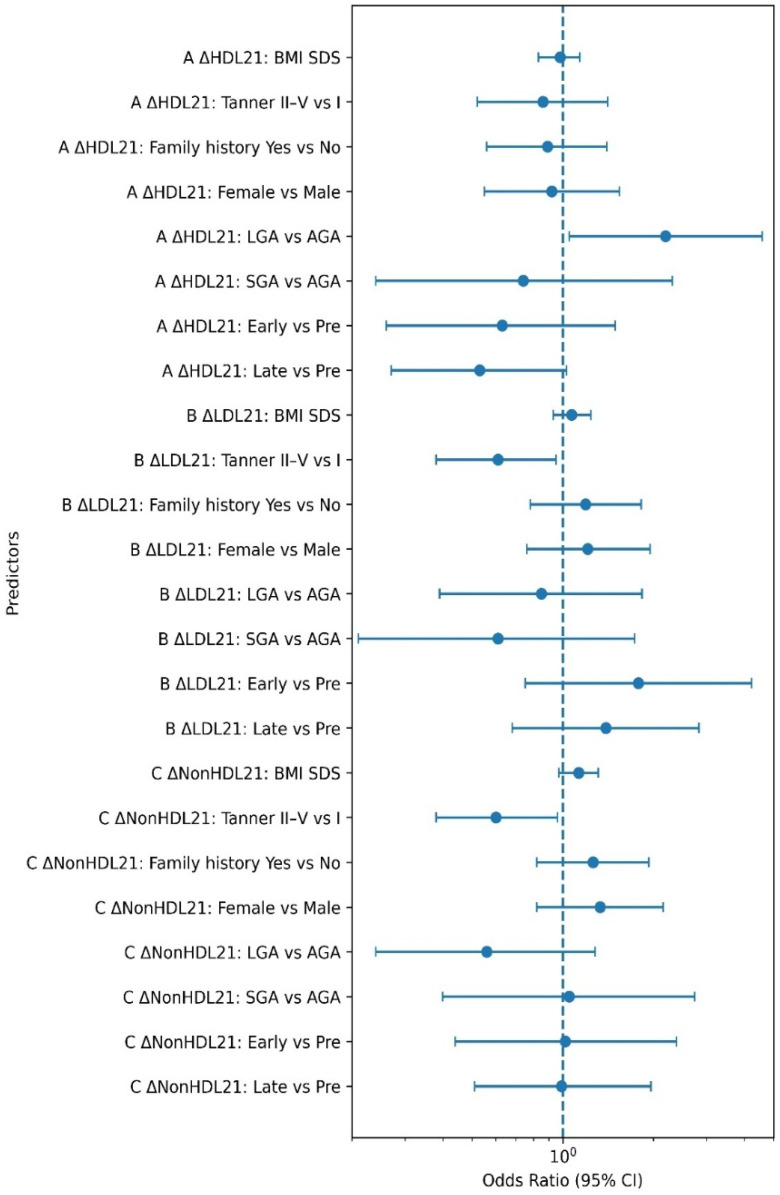
Forest plot of multivariable logistic regression analysis for predictors of lipid improvement. Odds ratios (ORs) and 95% confidence intervals (CIs) are shown for predictors of improvement in (A) HDL cholesterol, (B) LDL cholesterol, and (C) non-HDL cholesterol between baseline (T1) and first follow-up (T2). The vertical dashed line indicates an OR of 1. Models were adjusted for sex, BMI SDS, family history of dyslipidemia, pandemic period group, Tanner stage at baseline, and birth weight category. Improvement defined as ≥5 mg/dL increase in HDL, ≥10 mg/dL decrease in LDL or non-HDL cholesterol.

The associations between clinical factors and lipid improvement are visualized in Figure 2. The forest plot illustrates odds ratios and 95% confidence intervals for predictors of HDL, LDL, and non-HDL cholesterol improvement. Pubertal stage was associated with reduced likelihood of LDL and non-HDL cholesterol improvement, whereas large-for-gestational-age birth was associated with increased likelihood of HDL cholesterol improvement. Detailed analyses of lipid classification, additional metabolic markers, and full linear mixed model outputs are provided in the Supplementary Materials.

## 4. Discussion

In this longitudinal real-world cohort study of children and adolescents newly diagnosed with dyslipidemia, repeated lifestyle education delivered during routine outpatient follow-up was associated with improvements in several lipid parameters over time. In addition, developmental stage and early-life metabolic characteristics influenced the likelihood of lipid improvement. These findings provide clinically relevant insights into the role of sustained lifestyle counseling in the management of pediatric dyslipidemia in everyday clinical practice.

One of the principal findings of this study was the significant reduction in BMI SDS, total cholesterol, and non-HDL cholesterol during follow-up. Lifestyle modification is widely recommended as the first-line therapeutic approach for pediatric dyslipidemia, and our findings support the effectiveness of this strategy in routine clinical care [7,8]. The observed reduction in BMI SDS is consistent with prior evidence that lifestyle-based weight management interventions in children can yield improvements in both anthropometric and cardiometabolic outcomes, including in children with metabolic comorbidities [17]. In real-world practice, lifestyle counseling is typically reinforced repeatedly across multiple outpatient visits rather than delivered as a single structured intervention. The present results suggest that repeated reinforcement of dietary and physical activity recommendations may contribute to gradual metabolic improvements over time. In particular, reductions in total cholesterol and non-HDL cholesterol are clinically meaningful, as non-HDL cholesterol is increasingly recognized as an important marker of atherogenic lipid burden and long-term cardiovascular risk in pediatric populations [18]. We acknowledge, however, that the absolute magnitudes of improvement observed in this study—mean reductions of approximately 2.5 mg/dL in total cholesterol at T2 and 5.2 mg/dL at T3, and 2.0 mg/dL in non-HDL cholesterol at T2 and 4.1 mg/dL at T3—are modest and do not in themselves constitute sufficient cardiovascular risk reduction. These thresholds were defined as conservative, exploratory markers of early metabolic response to lifestyle education, not as targets for cardiovascular event prevention. Pharmacologic therapy is considered in our clinic when LDL cholesterol persistently remains ≥190 mg/dL (or ≥160 mg/dL with additional cardiovascular risk factors) despite at least 6 months of adequate lifestyle modification, consistent with current international and Korean national guidelines [7,8]. The observed early improvements should therefore be interpreted as indicators of treatment responsiveness, with cumulative cardiovascular benefit potentially accruing over longer periods of sustained lifestyle engagement, as supported by longitudinal epidemiological data linking childhood lipid levels to adult cardiovascular outcomes [4,5].

Longitudinal analysis using linear mixed models further demonstrated significant time-dependent reductions in total cholesterol and non-HDL cholesterol, while HDL cholesterol showed relatively stable patterns during follow-up. These findings suggest that lifestyle interventions may preferentially influence atherogenic lipid fractions rather than HDL cholesterol levels. Previous pediatric studies have reported variable effects of lifestyle modification on HDL cholesterol, likely reflecting the complex biological regulation of HDL metabolism [15,16]. Factors including genetic influences, habitual physical activity, and metabolic status may contribute to the relatively modest changes observed in HDL cholesterol in this cohort.

AST levels remained within normal laboratory reference ranges across all groups and time points. Because this study did not include liver imaging or hepatic clinical assessment, and given that AST was not a pre-specified outcome of this lipid-focused study, AST results are reported in Supplementary Table S6 rather than as a primary analytical outcome.

A second key finding of this study was the influence of developmental stage on metabolic response to lifestyle intervention. Logistic regression analysis revealed that pubertal stage was associated with a lower likelihood of LDL cholesterol and non-HDL cholesterol improvement. Puberty is accompanied by substantial hormonal and metabolic changes, including alterations in insulin sensitivity and lipid metabolism [9,10]. These physiological changes may partly explain why improvements in atherogenic lipid fractions were less pronounced among participants in more advanced pubertal stages. These findings suggest that earlier lifestyle intervention, particularly before or during early puberty, may provide greater potential for metabolic improvement.

In addition to developmental stage, early-life factors also appeared to influence metabolic responsiveness. Large-for-gestational-age birth was associated with an increased likelihood of HDL cholesterol improvement during follow-up. Birth weight has been increasingly recognized as a marker of early metabolic programming, reflecting intrauterine and early postnatal growth conditions that may influence long-term metabolic regulation [19,20]. Although the biological mechanisms underlying this association remain unclear, these findings suggest that early-life growth patterns may influence metabolic adaptation to lifestyle interventions later in childhood. Importantly, baseline HDL cholesterol levels did not differ significantly across birth weight categories (*p* = 0.277), and LGA participants had numerically lower baseline HDL (52.9 mg/dL) compared with AGA (56.6 mg/dL) and SGA (56.0 mg/dL) participants, arguing against the possibility that regression to the mean or higher baseline levels explained the LGA group’s greater HDL improvement. Developmental programming mechanisms, including epigenetic modifications associated with intrauterine overgrowth, may influence long-term adipokine secretion, lipid metabolism, and responsiveness to lifestyle modification [11,13]. In contrast, SGA birth was not a significant predictor of LDL (OR = 1.037, 95% CI 0.383–2.810, *p* = 0.943) or non-HDL cholesterol improvement (OR = 1.048, 95% CI 0.365–3.007, *p* = 0.931) in this cohort. SGA children who fail to achieve adequate postnatal catch-up growth are known to be at risk for insulin resistance, dyslipidemia, and increased cardiovascular risk—a pattern consistent with the developmental origins of metabolic disease [19]. However, because the present study excluded patients receiving growth hormone therapy, the catch-up growth status of SGA participants could not be determined, and whether the absence of a significant treatment response signal in SGA children reflects biological differences or a type II error due to the small subgroup size (*n* = 21) cannot be established. Future prospective studies with longitudinal growth trajectory data and larger SGA samples are warranted to address this question.

Participants in this study were categorized into three groups corresponding to distinct phases of the COVID-19 pandemic in Korea, each characterized by different behavioral and environmental conditions that may have influenced the metabolic context in which lifestyle education was delivered. Participants diagnosed during the pre-pandemic period (2019) received lifestyle education under baseline behavioral conditions, prior to any pandemic-related disruptions to physical activity or dietary patterns. Those diagnosed during the early-pandemic period (2020–June 2021) were exposed to nationwide school closures beginning in March 2020, strict social distancing mandates, and abrupt disruptions to organized physical activity—conditions that have been associated with marked reductions in children’s moderate-to-vigorous physical activity, increased sedentary behavior, and adverse dietary changes both in Korea and globally [21,22]. Korean-specific data indicate a significant increase in sedentary screen time during this period, even as overall physical activity levels showed only modest aggregate changes [22]. Participants diagnosed during the late-pandemic/recovery period (July 2021–2024) received lifestyle education during a phase of gradual behavioral normalization, corresponding to the staged resumption of in-person schooling and relaxation of public health restrictions; however, pandemic-related lifestyle disruptions—including elevated sedentary behavior and reduced organized sports participation—have been shown to persist beyond the formal relaxation of restrictions in Korean adolescents [23]. These phase-specific differences in behavioral context may have contributed to the heterogeneous metabolic trajectories observed across the three groups, particularly for parameters sensitive to lifestyle behaviors such as triglycerides and HDL cholesterol. Nonetheless, because direct measurement of physical activity, dietary intake, and other lifestyle behaviors was not available in this retrospective cohort, causal attribution is not possible and these interpretations remain speculative.

This study has several strengths. First, it included a relatively large clinical cohort of children and adolescents with dyslipidemia who were followed longitudinally in a real-world outpatient setting. Second, the use of linear mixed models allowed evaluation of metabolic trajectories while appropriately accounting for repeated measurements obtained at varying follow-up intervals. Third, the study simultaneously evaluated both longitudinal metabolic changes and predictors of lipid improvement, providing complementary insights into treatment responsiveness.

Nevertheless, several limitations should be considered. First, this study was retrospective and conducted at a single tertiary referral center; accordingly, the findings may not be generalizable to community-based or population-representative pediatric cohorts. Second, because no contemporaneous non-intervention control group was available—as all eligible patients at our clinic received lifestyle education per standard care—causal attribution of the observed metabolic changes to the educational intervention cannot be established. Third, adherence to lifestyle recommendations was not systematically documented in this retrospective cohort, representing a major limitation in interpreting the mechanisms underlying the observed changes. Fourth, direct measurements of dietary intake and physical activity were not available, precluding assessment of specific behavioral mediators. Fifth, the time-period groups were substantially imbalanced in size (pre-pandemic *n* = 46, early-pandemic *n* = 54, late-pandemic *n* = 337), which limits statistical power for detecting group-specific effects; in particular, interaction effects involving the smaller groups should be interpreted with caution. Sixth, follow-up data were not available for all participants at each time point (T3: *n* = 239; T4: *n* = 111), with particularly high attrition in the early-pandemic group (T4: *n* = 6, 88.9% attrition); although linear mixed models accommodate missing data under the MAR assumption, systematic dropout related to unmeasured outcomes cannot be excluded. Regarding the pattern of attrition, sex distribution did not differ significantly between participants retained at T4 (*n* = 111) and those with only T1–T2 data available, suggesting that sex-based differential dropout is unlikely to represent a major source of bias. Socioeconomic status was not systematically collected in this retrospective cohort and could not be evaluated as a dropout predictor, which represents a further limitation. Seventh, although Tanner stage was assessed at every outpatient visit, only the baseline value (T1) was used as a time-invariant covariate in the statistical models. This decision was scientifically justified by the study’s primary focus on baseline pubertal status as a modifier of treatment response, and is consistent with standard practice in pediatric lipid intervention research [8,10]. Nonetheless, it should be acknowledged that this approach may not fully capture the influence of ongoing pubertal progression on longitudinal lipid trajectories, and prospective studies incorporating time-updated pubertal assessments would be valuable. Eighth, the female predominance in this cohort (65.7%) likely reflects referral patterns at a tertiary pediatric endocrinology clinic, where girls are more frequently referred for pubertal and metabolic concerns, and may limit generalizability to male-predominant populations. Ninth, the multiple outcome variables examined in Table 3 were not corrected for across-variable multiplicity; findings should therefore be interpreted as exploratory and hypothesis-generating. Tenth, fasting compliance was assessed by patient or caregiver report at each visit rather than by objective verification; although participants who did not meet the fasting requirement were rescheduled for repeat sampling, the possibility of suboptimal fasting in a small number of cases cannot be completely excluded, and this represents a potential source of measurement variability in the lipid data.

## 5. Conclusions

In conclusion, repeated lifestyle education delivered during routine outpatient follow-up was associated with improvements in lipid profiles among children and adolescents with dyslipidemia. Developmental stage and early-life metabolic characteristics influenced the likelihood of lipid improvement, underscoring the value of sustained lifestyle counseling tailored to each child’s developmental and early-life background.

## Figures and Tables

**Table 1:** Baseline Characteristics of Study Participants.

Variable	Total (*n* = 437)	Pre-Pandemic (*n* = 46)	Early-Pandemic (*n* = 54)	Late-Pandemic (*n* = 337)	*p*-Value
Age at diagnosis, months	103.7 ± 20.7	102.2 ± 16.4	108.0 ± 23.4	103.3 ± 20.8	0.260
Bone age at diagnosis, years	9.07 ± 2.44	9.52 ± 1.93	9.81 ± 2.14	8.89 ± 2.52	0.020
Female sex, *n* (%)	287 (65.7)	38 (82.6)	45 (83.3)	204 (60.5)	<0.001
Family history of dyslipidemia, *n* (%)	261 (59.7)	29 (63.0)	41 (75.9)	191 (56.7)	0.030
Birth weight, kg	3.14 ± 0.49	—	—	—	—
SGA, *n* (%)	21 (4.8)	4 (8.7)	5 (9.3)	12 (3.6)	0.260
LGA, *n* (%)	35 (8.0)	3 (6.5)	5 (9.3)	27 (8.0)	
BMI, kg/m^2^	19.02 ± 3.78	19.18 ± 4.12	18.62 ± 3.51	19.07 ± 3.77	0.690
BMI SDS	0.61 ± 1.48	0.72 ± 1.73	0.43 ± 1.53	0.62 ± 1.44	0.600
Tanner stage II–V, *n* (%)	271 (62.3)	32 (71.1)	38 (70.4)	201 (59.8)	0.150
Total cholesterol, mg/dL	198.1 ± 24.6	203.0 ± 27.5	201.0 ± 23.2	197.0 ± 24.4	0.200
Abnormal TC ≥ 200 mg/dL, *n* (%)	209 (47.8)	25 (54.3)	30 (55.6)	154 (45.7)	0.070
Triglyceride, mg/dL	121.2 ± 73.9	122.9 ± 68.5	130.7 ± 85.7	119.4 ± 72.6	0.570
HDL cholesterol, mg/dL	56.3 ± 13.0	57.2 ± 15.8	58.7 ± 12.1	55.8 ± 12.8	0.270
LDL cholesterol, mg/dL	124.4 ± 24.4	123.8 ± 25.7	129.5 ± 26.9	123.7 ± 23.8	0.260
Non-HDL cholesterol, mg/dL	141.9 ± 23.3	145.8 ± 26.5	142.3 ± 26.0	141.2 ± 22.4	0.460

Values are presented as mean ± SD or *n* (%). *p*-values were calculated using ANOVA for continuous variables and chi-square tests for categorical variables. Groups correspond to COVID-19 pandemic phases defined in the Methods: Pre-pandemic (2019), Early-pandemic (2020–June 2021), and Late-pandemic (July 2021–2024). SGA: small for gestational age; LGA: large for gestational age; BMI SDS: body mass index standard deviation score; TC: total cholesterol; HDL: high-density lipoprotein; LDL: low-density lipoprotein.

**Table 2:** Changes in Anthropometric and Laboratory Parameters During Follow-up.

Variable	Change T2–T1 (Mean ± SD)	*p*-Value	Change T3–T1 (Mean ± SD)	*p*-Value
BMI SDS	−0.11 ± 0.38	<0.001	−0.09 ± 0.50	0.010
Total cholesterol, mg/dL	−2.54 ± 21.99	0.020	−5.15 ± 21.46	<0.001
Triglyceride, mg/dL	−3.39 ± 85.62	0.410	−6.15 ± 81.83	0.250
HDL cholesterol, mg/dL	−0.50 ± 8.33	0.210	−1.09 ± 9.05	0.060
LDL cholesterol, mg/dL	−1.58 ± 20.87	0.110	−2.64 ± 23.03	0.080
Non-HDL cholesterol, mg/dL	−2.04 ± 19.48	0.030	−4.05 ± 20.38	<0.001

Changes represent differences from baseline (T1). Paired *t*-tests were used.

**Table 3:** Fixed Effects from Linear Mixed Models.

Outcome	Time Effect (*p*)	Group Effect (*p*)	Time × Group Interaction (*p*)
Total cholesterol	0.011	0.660	0.310
Triglyceride	0.280	0.680	0.650
HDL cholesterol	0.380	0.037	0.250
LDL cholesterol	0.450	0.230	0.490
Non-HDL cholesterol	0.037	0.910	0.260
TSH	0.014	0.440	0.300

Linear mixed models were adjusted for sex, family history, birth weight category, and Tanner stage at baseline as time-invariant covariates, and BMI SDS at each visit as a time-varying covariate. REML estimation with unstructured (UN) covariance matrix was applied for all outcomes. A *p*-value < 0.05 was considered statistically significant. AST results are presented in Supplementary Table S6.

**Table 4:** Predictors of Lipid Improvement Between T1 and T2.

Variable	HDL Improvement OR (95% CI)	*p*-Value	LDL Improvement OR (95% CI)	*p*-Value	Non-HDL Improvement OR (95% CI)	*p*-Value
Tanner stage II–V	0.86 (0.52–1.41)	0.550	0.61 (0.38–0.95)	0.030	0.60 (0.38–0.96)	0.030
LGA birth	2.19 (1.05–4.59)	0.040	0.85 (0.39–1.83)	0.680	0.56 (0.24–1.28)	0.170

Improvement defined as ≥5 mg/dL increase in HDL, ≥10 mg/dL decrease in LDL or non-HDL cholesterol. Adjusted for sex, BMI SDS, family history of dyslipidemia, pandemic period group, Tanner stage, and birth weight category.

## Data Availability

The data presented in this study are available from the corresponding author upon reasonable request. The data are not publicly available due to institutional privacy and ethical restrictions.

## Supplementary Materials

The following supporting information can be downloaded at: https://www.mdpi.com/article/10.3390/children13050682/s1, Table S1: Detailed Baseline Distribution of Lipid Categories; Table S2: Linear Mixed Model Parameter Estimates (Estimated Marginal Means ± SE) - Excluding AST; Table S3: Longitudinal Changes in Additional Metabolic Markers; Table S4: Multivariable Logistic Regression Analysis for Lipid Improvement Using Percentage Change Criteria; Table S5: Persistence of Lipid Improvement at T4 Among Participants with T2 Improvement; Table S6: AST Estimated Marginal Means ± SE - Compound Symmetry (CS) Covariance Structure; Table S7: Number of participants with available data at each follow-up visit, stratified by pandemic-period group.
