# Supplementary material for: Longitudinal Effectiveness of Repeated Lifestyle Education in Pediatric Dyslipidemia: Developmental and Environmental Modifiers in a Real-World Clinical Cohort
Source: Children (Basel). 2026 May 16;13(5):682. doi: 10.3390/children13050682 (PMC13204048; doi:10.3390/children13050682)
Supplement: Supplementary file 1 [file children-13-00682-s001.zip › children-4286169-supplementary.pdf]

Supplementary Materials

Longitudinal Effectiveness of Repeated Lifestyle Education in Pediatric Dyslipidemia: Developmental and Environmental Modifiers in a Real-World Clinical Cohort

S1. Baseline Distribution of Lipid Categories

The detailed distribution of lipid classification categories is presented in Supplementary Table S1. The proportions of participants classified as normal, borderline, or abnormal for total cholesterol, triglycerides, HDL cholesterol, and LDL cholesterol did not differ significantly across time-period groups.

A trend toward a higher proportion of abnormal total cholesterol was observed in the earlier time periods, but this did not reach statistical significance.

Table S1. Detailed Baseline Distribution of Lipid Categories

Variable	Category	Total (n=437)	Pre-pandemic (n=46)	Early-pandemic (n=54)	Late-pandemic (n=337)	p-value
Total cholesterol	Normal (<170 mg/dL)	42 (9.6)	6 (13.0)	0 (0.0)	36 (10.7)	0.070
	Borderline (170–199 mg/dL)	186 (42.6)	15 (32.6)	24 (44.4)	147 (43.6)	
	Abnormal (≥200 mg/dL)	209 (47.8)	25 (54.3)	30 (55.6)	154 (45.7)	
Triglycerides	Normal	142 (32.5)	16 (34.8)	13 (24.1)	113 (33.5)	0.620
	Borderline	82 (18.8)	7 (15.2)	13 (24.1)	62 (18.4)	
	Abnormal	213 (48.7)	23 (50.0)	28 (51.9)	162 (48.1)	
HDL cholesterol	Normal	347 (79.4)	35 (76.1)	48 (88.9)	264 (78.3)	0.250
	Borderline	53 (12.1)	8 (17.4)	2 (3.7)	43 (12.8)	
	Abnormal	37 (8.5)	3 (6.5)	4 (7.4)	30 (8.9)	
LDL cholesterol	Normal	112 (25.6)	12 (26.1)	10 (18.5)	90 (26.7)	0.450
	Borderline	153 (35.0)	19 (41.3)	18 (33.3)	116 (34.4)	
	Abnormal	172 (39.4)	15 (32.6)	26 (48.1)	131 (38.9)	

Values are presented as n (%). p-values were calculated using chi-square tests.

Table S1b. Baseline HDL Cholesterol Levels by Birth Weight Category

Birth weight category	n	Baseline HDL cholesterol (mg/dL)
SGA	21	56.0 ± 14.9
AGA	381	56.6 ± 13.0
LGA	35	52.9 ± 12.2
**p-value (one-way ANOVA)**	—	0.277

Values are presented as mean ± SD (mg/dL). SGA: small-for-gestational-age; AGA: appropriate-for-gestational-age; LGA: large-for-gestational-age. The LGA group had numerically lower baseline HDL cholesterol than the AGA group (52.9 vs 56.6 mg/dL), arguing against the possibility that higher baseline HDL levels explained the greater HDL improvement observed in the LGA group.

S2. Full Linear Mixed Model Output

Full parameter estimates and estimated marginal means for all outcome variables are presented in Supplementary Table S2 (excluding AST; AST results are presented in Table S6).

These analyses confirmed significant time-dependent reductions in total cholesterol, non-HDL cholesterol, and TSH. HDL cholesterol demonstrated a significant between-group difference that was independent of time.

Table S2. Linear Mixed Model Parameter Estimates (Estimated Marginal Means ± SE) — Excluding AST

Outcome	Timepoint	Pre-pandemic	Early-pandemic	Late-pandemic
Total cholesterol (mg/dL)	T1	199.9 ± 4.0	195.9 ± 3.7	194.9 ± 2.2
	T2	198.4 ± 4.3	192.2 ± 4.0	192.0 ± 2.3
	T3	194.6 ± 4.4	186.5 ± 4.5	191.0 ± 2.4
	T4	189.7 ± 5.5	201.5 ± 8.5	192.0 ± 3.0
Triglycerides (mg/dL)	T1	120.4 ± 11.9	130.9 ± 10.9	117.7 ± 6.3
	T2	113.2 ± 12.5	120.8 ± 11.5	117.4 ± 6.4
	T3	109.4 ± 12.6	108.0 ± 13.3	115.1 ± 6.7
	T4	107.6 ± 17.8	87.9 ± 31.0	126.5 ± 9.8
HDL cholesterol (mg/dL)	T1	56.4 ± 2.0	57.8 ± 1.8	55.1 ± 1.1
	T2	57.8 ± 1.9	56.3 ± 1.8	54.1 ± 1.1
	T3	57.6 ± 2.0	54.6 ± 2.0	53.6 ± 1.1
	T4	57.8 ± 2.1	59.1 ± 3.0	53.3 ± 1.2
LDL cholesterol (mg/dL)	T1	122.0 ± 4.1	125.7 ± 3.7	122.6 ± 2.3
	T2	122.1 ± 4.1	124.7 ± 3.8	120.7 ± 2.3
	T3	124.1 ± 4.7	121.9 ± 4.8	120.2 ± 2.5
	T4	121.8 ± 5.1	136.9 ± 7.6	120.5 ± 2.8
Non-HDL cholesterol (mg/dL)	T1	143.7 ± 4.2	138.2 ± 3.9	139.9 ± 2.7
	T2	140.9 ± 4.3	136.2 ± 4.0	138.0 ± 2.8
	T3	137.2 ± 4.7	131.9 ± 4.7	137.5 ± 2.9
	T4	131.9 ± 5.3	141.5 ± 7.7	138.7 ± 3.3
TSH (µU/mL)	T1	2.7 ± 0.2	2.4 ± 0.2	2.6 ± 0.1
	T2	2.3 ± 0.2	2.3 ± 0.2	2.5 ± 0.1
	T3	2.0 ± 0.2	2.2 ± 0.3	2.5 ± 0.1
	T4	2.1 ± 0.3	2.6 ± 0.4	2.4 ± 0.2

Models were adjusted for sex, family history of dyslipidemia, and birth weight category (time-invariant), Tanner stage at baseline (time-invariant), and BMI SDS at each visit (time-varying). Unstructured (UN) covariance matrix applied. AST results are presented in Table S6.

S3. Longitudinal Changes in Additional Metabolic Markers

Supplementary Table S3 presents the longitudinal changes in additional metabolic markers, including TG/HDL ratio, fasting glucose, TSH, AST, and ALT.

The TG/HDL ratio showed a decreasing trend during follow-up, but this did not reach statistical significance. Fasting glucose demonstrated a modest increase at later follow-up visits, though the clinical significance was limited. TSH showed a significant reduction, consistent with the linear mixed model findings.

Table S3. Longitudinal Changes in Additional Metabolic Markers

Variable	Change T2–T1 (Mean ± SD)	p-value	Change T3–T1 (Mean ± SD)	p-value
TG/HDL ratio	−0.12 ± 0.85	0.180	−0.15 ± 0.92	0.130
Fasting glucose (mg/dL)	0.06 ± 15.13	0.940	2.17 ± 15.36	0.030
TSH (µU/mL)	−0.15 ± 1.35	0.020	−0.22 ± 1.19	0.010
AST (U/L)	−1.43 ± 10.13	<0.001	−0.93 ± 16.46	0.380
ALT (U/L)	−0.89 ± 12.89	0.150	−0.69 ± 10.62	0.310

Changes represent differences from baseline (T1). Paired t-tests were used.

S4. Sensitivity Analysis Using Percentage-Change Lipid Improvement Criteria

To evaluate the sensitivity of the lipid improvement criteria, logistic regression analyses were repeated using percentage-change definitions (HDL increase ≥10%, LDL decrease ≥10%, non-HDL decrease ≥10%) in place of the absolute-change criteria (HDL increase ≥5 mg/dL, LDL decrease ≥10 mg/dL, non-HDL decrease ≥10 mg/dL), with the same set of covariates.

Results for HDL and LDL were directionally consistent with the primary analysis. LGA birth was a significant predictor of HDL improvement ≥10% (OR=2.461, 95% CI 1.171–5.172, p=0.018), and Tanner stage II–V was a significant predictor of LDL reduction ≥10% (OR=0.622, 95% CI 0.388–0.997, p=0.048). For non-HDL, no significant predictor was identified under the 10% threshold (Tanner stage p=0.205), indicating that the primary analysis finding for non-HDL (Tanner stage OR=0.60, p=0.030) is sensitive to the choice of improvement criterion. Accordingly, this finding should be interpreted with caution as an exploratory result, as also noted in the main manuscript.

Table S4. Multivariable Logistic Regression Analysis for Lipid Improvement Using Percentage-Change Criteria

Variable	HDL ≥10% increase OR (95% CI)	p-value	LDL ≥10% decrease OR (95% CI)	p-value	Non-HDL ≥10% decrease OR (95% CI)	p-value
BMI SDS	1.087 (0.921–1.283)	0.325	1.097 (0.942–1.278)	0.232	1.095 (0.933–1.285)	0.265
Sex: female vs. male	0.921 (0.536–1.585)	0.767	1.175 (0.721–1.917)	0.518	1.324 (0.790–2.220)	0.287
Family history of dyslipidemia: yes vs. no	0.860 (0.534–1.387)	0.537	1.332 (0.856–2.072)	0.204	1.154 (0.729–1.827)	0.541
Tanner II–V vs I	0.800 (0.473–1.353)	0.405	0.622 (0.388–0.997)	**0.048***	0.726 (0.442–1.192)	0.205
LGA vs AGA	2.461 (1.171–5.172)	**0.018***	0.941 (0.429–2.063)	0.879	0.827 (0.357–1.916)	0.658
SGA vs AGA	1.002 (0.321–3.125)	0.997	1.037 (0.383–2.810)	0.943	1.048 (0.365–3.007)	0.931
Early-pandemic vs pre-pandemic	0.713 (0.275–1.845)	0.485	1.070 (0.426–2.688)	0.886	0.803 (0.299–2.159)	0.664
Late-pandemic vs pre-pandemic	0.719 (0.346–1.496)	0.378	1.376 (0.661–2.865)	0.394	1.334 (0.626–2.843)	0.455

*OR: odds ratio; CI: confidence interval. *p < 0.05. Adjusted for sex, BMI SDS, family history of dyslipidemia, time period of diagnosis, Tanner stage at baseline, and birth weight category. Male (ref.), No family history of dyslipidemia (ref.), Tanner stage I (ref.), AGA (ref.), Pre-pandemic (ref.)*

S5. Persistence of Lipid Improvement at T4 Among Participants with T2 Improvement

To assess whether lipid improvement at the first follow-up visit (T2) was sustained through the third follow-up visit (T4), cross-tabulation analyses were conducted among participants with available T4 data (n=111).

Among participants who showed improvement at T2, the proportions maintaining improvement at T4 were 65.5% (19/29) for HDL, 58.6% (17/29) for LDL, and 66.7% (18/27) for non-HDL — approximately three times higher than rates among those without T2 improvement (19.5%, 17.1%, and 22.6%, respectively). These findings suggest that lipid improvement at the first follow-up visit is meaningfully associated with sustained improvement at later time points.

Table S5a. Persistence of HDL Cholesterol Improvement (n=111)

	No improvement at T4	Improvement at T4	Total
No improvement at T2	66 (80.5%)	16 (19.5%)	82 (100%)
**Improvement at T2**	10 (34.5%)	**19 (65.5%)**	29 (100%)
Total	76 (68.5%)	35 (31.5%)	111 (100%)

Table S5b. Persistence of LDL Cholesterol Improvement (n=111)

	No improvement at T4	Improvement at T4	Total
No improvement at T2	68 (82.9%)	14 (17.1%)	82 (100%)
**Improvement at T2**	12 (41.4%)	**17 (58.6%)**	29 (100%)
Total	80 (72.1%)	31 (27.9%)	111 (100%)

Table S5c. Persistence of Non-HDL Cholesterol Improvement (n=111)

	No improvement at T4	Improvement at T4	Total
No improvement at T2	65 (77.4%)	19 (22.6%)	84 (100%)
**Improvement at T2**	9 (33.3%)	**18 (66.7%)**	27 (100%)
Total	74 (66.7%)	37 (33.3%)	111 (100%)

Improvement defined as HDL increase ≥10%, LDL decrease ≥10%, or non-HDL decrease ≥10% (percentage-change criteria, consistent with Supplementary Table S4). Data available at T4: n=111.

S6. AST Estimated Marginal Means from Linear Mixed Model (Compound Symmetry Covariance Structure)

AST (aspartate aminotransferase) was not a pre-specified outcome of this study. The Unstructured (UN) covariance structure produced non-convergent variance estimates for AST (SE ≈ mean; 95% CI extending into physiologically implausible negative values; ΔAIC UN vs. CS = −237.5), indicating severe model overparameterization. Accordingly, a Compound Symmetry (CS) covariance structure was applied for AST only, yielding stable and clinically plausible SE estimates (1.49–4.25 U/L).

Estimated marginal mean AST values across all groups and time points remained within normal laboratory reference ranges (21–39 U/L; upper limit of normal <40 U/L). As no liver imaging or clinical hepatic assessment was performed in this study, these findings should not be interpreted as evidence of clinically meaningful liver pathology.

Table S6. AST Estimated Marginal Means ± SE — Compound Symmetry (CS) Covariance Structure

Timepoint	Pre-pandemic	Early-pandemic	Late-pandemic	p-value (fixed effects)
T1	27.4 ± 2.1	32.5 ± 2.0	26.3 ± 1.5	Time p=0.001<br>Group p<0.001<br>Time×Group p=0.002
T2	30.5 ± 2.1	30.4 ± 2.0	24.5 ± 1.5	
T3	30.9 ± 2.3	39.1 ± 2.4	23.9 ± 1.6	
T4	29.2 ± 2.6	21.0 ± 4.2	23.0 ± 1.8	

Values are estimated marginal means ± SE (U/L). Compound Symmetry (CS) covariance structure applied. Adjusted for sex, family history of dyslipidemia, and birth weight category (time-invariant), Tanner stage at baseline (time-invariant), and BMI SDS at each visit (time-varying). Post-hoc pairwise comparisons among the three pandemic-period groups were adjusted using the Bonferroni method. All estimated values are within the normal laboratory reference range (<40 U/L).

Table S7. Number of Participants with Available Data at Each Follow-Up Visit, Stratified by Pandemic-Period Group

Group	T1	T2	T3	T4
Pre-pandemic	46	46	31	23
Early-pandemic	54	54	25	6
Late-pandemic	337	337	183	82
**Total**	437	437	239	111

T1, baseline visit; T2, first follow-up; T3, second follow-up; T4, third follow-up. Attrition was particularly high in the early-pandemic group at T4 (n = 6; 88.9% attrition), reflecting the shorter follow-up period available for patients diagnosed during 2020–June 2021. Linear mixed models were estimated under the missing-at-random (MAR) assumption, which allows all participants with at least one available observation to contribute to the analysis.
